# Treatment of canine oral squamous cell carcinomas with photodynamic therapy

**DOI:** 10.1054/bjoc.1999.1094

**Published:** 2000-03-06

**Authors:** D L McCaw, E R Pope, J T Payne, M K West, R V Tompson, D Tate

**Affiliations:** College of Veterinary Medicine, College of Engineering, University of Missouri, Columbia, MO, 65211, USA

**Keywords:** photodynamic therapy, squamous cell carcinoma, canine, photochlor, HPPH

## Abstract

Eleven dogs with naturally occurring oral squamous cell carcinomas were treated with photodynamic therapy (PDT) using Photochlor (HPPH) as the photosensitizer. The largest length of the tumours measured in a two-dimensional plane ranged from 0.9 to 6.8 cm. Seven of the tumours invaded underlying bone as determined by radiograph appearance. Photochlor was injected intravenously at a dose of 0.3 mg kg^–1^. Forty-eight hours later the tumours were treated. Tumours with a surface to base depth of greater than 1 cm were surgically reduced to less than 1 cm. Irradiation with 665 nm light with an energy density of 100 J cm^–2^was administered. Eight dogs were considered cured with no tumour recurrence for at least 17 months after treatment. Local treatment of oral squamous cell carcinomas with PDT appears to give results similar to those obtained with surgical removal of large portions of the mandible or maxilla. The cosmetic results with PDT are superior to those of radical surgical removal. The new sensitizer, Photochlor, appears effective for oral squamous carcinomas with results similar to those reported for other sensitizers. © 2000 Cancer Research Campaign


*British Journal of Cancer* (2000) **82**(7), 1297–1299
© 2000 Cancer Research Campaign
DOI: 10.1054/ bjoc.1999.1094, available online at http://www.idealibrary.com on IDEAL®

### Treatment of canine oral squamous cell carcinomas with photodynamic therapy

**DL McCaw[1], ER Pope[1], JT Payne[1], MK West[1], RV Tompson[2], and D Tate[1]**

[1]College of Veterinary Medicine and [2]College of Engineering, University of Missouri, Columbia, MO 65211, USA

**Summary** Eleven dogs with naturally occurring oral squamous cell carcinomas were treated with photodynamic therapy (PDT) using Photochlor (HPPH) as the photosensitizer. The largest length of the tumours measured in a two-dimensional plane ranged from 0.9 to 6.8 cm. Seven of the tumours invaded underlying bone as determined by radiograph appearance. Photochlor was injected intravenously at a dose of 0.3 mg $kg^{-1}$. Forty-eight hours later the tumours were treated. Tumours with a surface to base depth of greater than 1 cm were surgically reduced to less than 1 cm. Irradiation with 665 nm light with an energy density of 100 J $cm^{-2}$ was administered. Eight dogs were considered cured with no tumour recurrence for at least 17 months after treatment. Local treatment of oral squamous cell carcinomas with PDT appears to give results similar to those obtained with surgical removal of large portions of the mandible or maxilla. The cosmetic results with PDT are superior to those of radical surgical removal. The new sensitizer, Photochlor, appears effective for oral squamous carcinomas with results similar to those reported for other sensitizers. © 2000 Cancer Research Campaign

**Keywords**: photodynamic therapy; squamous cell carcinoma; canine; photochlor; HPPH

Photodynamic therapy (PDT) for the treatment of cancerous and non-cancerous lesions is becoming an accepted therapy. The process involves the presence of a photoactive agent which is activated in the target tissue by non-thermal light resulting in necrosis. Porfimer sodium (Photofrin, Quadra Logic Technologies) is the only commercially available sensitizer in the USA. Prolonged skin photosensitivity lasting as long as 3 months in some cases and activation at 630 nm which limits the depth of light penetration in tissue has resulted in the search for other photosensitizing compounds. One such agent that is now in phase I/II trials is pyropheophorbide-alpha-hexyl-ether (HPPH, Photochlor). HPPH is derived from pheophorbide-a obtained from *Spirrulina* algae. Potential advantages of HPPH over porfimer sodium are a major visible absorption peak at 665 nm allowing slightly increased tissue penetration and rapid clearance from normal skin in the murine model (Bellnier et al, 1993). If the rapid skin clearance occurs in other species, decreased skin photosensitivity might be the result. Previous work in dogs indicated a mean serum half-life is 26 h and no toxicity at an intravenous (i.v.) dose of 0.3 mg $kg^{-1}$ (Payne et al, 1996).

Tumours located where light can be easily applied such as the oral cavity are good candidates for PDT therapy. The ideal tumours to treat with PDT would be those that do not metastasize since no systemic anti-tumour effects are present with PDT. Dogs with gingival squamous cell carcinomas (SCC) are good candidates for PDT. Oral SCCs are locally aggressive with local bone invasion seen in 77% of cases. Only 10% of tumours spread to regional lymph nodes and 3% metastasize to the lungs (Head, 1990). Aggressive surgical therapy is the present recommended treatment for these tumours and can result in long remissions. Removal of large portions of the mandible can be cosmetically and aesthetically displeasing. This study was undertaken to treat naturally occurring oral SCCs in dogs with PDT using HPPH as the photosensitizing drug.

*Received 11 May 1999*
*Revised 8 November 1999*
*Accepted 11 November 1999*

*Correspondence to:* DL McCaw, Clydesdale Hall, 379 East Campus Drive, Columbia, MO 65211, USA

### MATERIALS AND METHODS

### Animals

Eleven client-owned dogs presented to the University of Missouri Veterinary Medical Teaching Hospital for treatment of oral SCC were used (Table 1). The clients were informed that aggressive surgery was the current accepted treatment for oral SCCs. The dogs were entered into the experimental study at the request of the owners. The ages ranged from 7 to 13 years. Nine dogs were females and two were males. The tumours had been detected 1–5 months prior to presentation and the tumours of four dogs had been locally excised during that period but had recurred. Physical, radiographic and laboratory examination indicated that neither spread of the tumour from the local area nor organ failure would limit the dog’s life. All tumours were histopathologically confirmed to be SCCs.

### Tumour staging

The tumours were staged as: T0 – no evidence of tumour; T1 – tumour < 2 cm maximum diameter; T2 – tumour 2–4 cm maximum diameter; T3 – tumour > 4 cm maximum diameter. All tumours were substaged as: a – without bone invasion; b – with bone invasion. Tumour staging for the 11 dogs is given in Table 1.

**Table 1** Animal information and outcome

| Gender | Age (years) | Tumour location | Time present before PDT (months) | Previous treatment | Tumour size* (cm) | Surgical debulking | Tumour remaining | Tumour stage | Outcome/ time to outcome (months) |
|---|---|---|---|---|---|---|---|---|---|
| Female (spayed) | 9 | Rostral left mandible | 1.5 | None | 2.4 × 1.6 | Yes | Yes | T2b | Died – no tumour recurrence (29) |
| Female (spayed) | 7 | Caudal right maxilla | 5.0 | Surgical removal | 2.2 × 1.6 | No | Yes | T2a | Died – no tumour recurrence (36) |
| Male | 10 | Middle right mandible | 2.5 | None | 6.8 × 5.4 | Yes | Yes | T3b | Tumour recurred (9) |
| Female | 10 | Rostral left mandible | 1.5 | None | 2.1 × 1.9 | Yes | No | T2a | Alive (49) |
| Female (spayed) | 8 | Rostral left mandible | 1.0 | Surgical removal | 0.9 × 0.7 | Yes | No | T1a | Alive (36) |
| Male (castrated) | 10 | Rostral left mandible | 2 | None | 2.3 × 1.8 | Yes | Yes | T2b | Died – no tumour recurrence (23) |
| Female (spayed) | 7 | Rostral left maxilla | 1 | Surgical removal | 1.1 × 1.0 | No | Yes | T1b | Alive (30) |
| Female (spayed) | 11 | Caudal left mandible | 3 | Surgical removal | 2.5 × 1.2 | No | Yes | T2a | Died – no tumour recurrence (17) |
| Female (spayed) | 10 | Rostral right mandible | 2 | None | 2.5 × 1.8 | Yes | Yes | T2b | Tumour remained – euthanatized (6) |
| Female (spayed) | 13 | Rostral bilateral maxilla | 1 | None | 2.7 × 2.5 | Yes | Yes | T2b | Alive (23) |
| Female (spayed) | 13 | Rostral left mandible | 1 | None | 2.4 × 2.1 | Yes | Yes | T2b | Tumour metastasis (3) |

*Caliper measurement of grossly estimated tumour margin: longest length × longest width perpendicular to length.

### Photosensitizer and light source

This study was performed under an investigational new animal drug (INAD) permit obtained from the United States Food and Drug Administration. The photosensitizer (Photochlor-HPPH) was kindly provided by Dr Thomas J Dougherty (Roswell Park Cancer Institute, Buffalo, NY, USA) in an aqueous solution (0.1% polysorbate 80, 2.0% ethanol, 5.0% dextrose in water). The dose was 0.3 mg $kg^{-1}$ which was injected i.v. 48 h prior to surgical debulking and irradiation.

An argon ion pumped dye laser (Coherent Laser Systems Innova 200 and 599 respectively, San Jose, CA, USA) tuned to 665 nm wavelength was used for the treatments. A quartz optic fibre with a sapphire microlens affixed at the distal end was used to ensure uniform light delivery at the surface of the patient (PDT Systems Inc., Santa Barbara, CA, USA) The laser and fibre output were monitored before and after each treated area with a power meter (Coherent, model 30 W).

### Anaesthesia and analgesia

All treatments were accomplished under general anaesthesia. The dogs were administered atropine, xylazine and morphine as pre-anaesthetic agents. Anaesthesia was induced with thiopental and maintained after tracheal intubation with isoflorane. Morphine was administered after anaesthetic recovery and for 18 h post-treatment.

### Treatment

Tumours that were thicker than 1 cm, as determined by measuring the tumour or extent of the tumour on radiographs, were surgically debulked just prior to irradiation to reduce the thickness to less than 1 cm with no intent to completely remove the neoplastic tissue. Tissue was obtained from the surgical bed and examined for tumour cells. Surgical debulking was performed in eight dogs, while three had tumours small enough that surgery was not necessary (Table 1). Of the eight dogs that had surgery, six dogs had histopathologic evidence of residual tumour. The specimens were too small in the other two for an interpretation. Any teeth that were loose were removed.

The tumours or surgical beds were irradiated with 665 nm wavelength light, a calculated incident light dose of 100 J $cm^{-2}$, and a fluence rate of 100 mW $cm^{-2}$. Each treated area was a circle described by the light spot from the optic fibre. The sapphire lens on the distal end of the fibre provided uniform power density within the illuminated spot. The tumour bed and approximately 3 mm of normal appearing tissue were then irradiated. A 3 cm spot was the largest that was used. The duration of the treatment for each treated area was 16 min, 40 s. Most tumours required more than one spot. In this case the spots were overlapped to avoid untreated areas. Tissue outside of the treatment field was shielded from incident laser light using moistened gauze and paper surgical drapes. The fibre was positioned so that light could be delivered nearly perpendicular to the tissue. Several spots were necessary for some dogs as a result of the contour of the tissue.

Varying degrees of swelling were observed after irradiation. This swelling resolved within 4 days. All dogs ate when offered food the day after treatment. Dogs were released to owners for follow-up care as early as 1 day post-treatment. The owners were instructed to avoid exposing the dogs to direct sunlight either indoors or outdoors for more than 15 min three times daily. The light restriction was to last for 4 weeks. No owners observed photophobia. No dog experienced a general reaction such as redness or swelling as has been described in humans. An area on the dorsal portion of the nose of one dog became ulcerated between 1 and 2 weeks post-treatment. The dog was housed outside after treatment. The lesion was healed by post-treatment week 4. Darkening of the skin on the ventral abdomen occurred in another dog.

### RESULTS (Table 1)

Normal teeth that were included in the treatment field became loose within 4 weeks of therapy. Most lesions healed within

 © 2000 Cancer Research Campaign

6 weeks but three dogs required either removal of a bone sequestrum or surgical debridement of devitalized bone to aid healing. One dog had no apparent benefit from PDT with recurrence or residual tumour present at 1 month after treatment. Metastatic disease in the regional lymph node was present in one dog 3 months after treatment, however there was no return of tumour in the treated area. One dog had confirmed recurrence and was euthanazed at 9 months after treatment. Four dogs died of unrelated causes and had no evidence of the oral SCC at 17, 23, 29 and 36 months after treatment. Four dogs were alive with no evidence of tumour recurrence at the time of writing 23, 30, 36 and 49 months after treatment. Median survival of the 11 treated dogs was 29 months.

### DISCUSSION

Gingival SCC is the second most common malignant neoplasm of the canine oral cavity. The tumours are aggressive with local bone invasion seen in 77% of cases. Loss of teeth due to bone destruction is common. Only 10% of tumours spread to regional lymph nodes and 3% metastasize to the lungs (Head, 1990). Local excision of the tumours is of little benefit with a median survival of 9 months and about 35% of dogs tumour-free at 1 year (Todoroff and Brodey, 1979). Aggressive surgery in the form of total or partial mandible resection results in a median survival of 15.8 months (Salisbury and Lantz, 1988). Radiation therapy will result in complete remission in 16% of tumours for 1 year. The current recommended therapy is resection of the mandible or maxilla to assure tumour-free surgical borders. Post-operative complications include prehension dysfunction, excessive drooling or salivation, and palatal ulceration secondary to malocclusion. For those tumours that cannot be surgically removed, radiation therapy is the treatment of choice. Radiation therapy results in mucositis. PDT is without the complications of surgery and radiation therapy and, because oral SCC do not readily metastasize, local therapy is potentially curable.

Previous work with PDT in SCCs suggests that it would be benefical in treatment of canine oral SCCs. A human pharyngeal SCC cell line has been shown to be one of the most sensitive cell types to PDT in vitro (Boonkitticharoen et al, 1997). Early-stage disease SCC of the head and neck treated with Photofrin resulted in 20 of 26 (77%) people achieving a complete response based on physical examination and histopathological confirmation. Sixteen people remained free of tumour for periods up to 51 months (Wenig et al, 1990). Using Photofrin in dogs for oral SCCs, the median remission was 13 months, with 11 of 14 dogs still alive at the time of writing (Beck, 1992).

The only other report on the usage of HPPH for naturally occurring tumours was treatment of SCC on the face in cats. It was found to be effective for early-stage disease (Magne et al, 1997). The results using other sensitizing agents for facial SCC in cats were similar (Peaston et al, 1993; Chang et al, 1998).

Our results were excellent for T1 and T2 disease and were comparable to surgery and better than radiation therapy. The cosmetic results were superior. Healing complications were minimal, however some lesions required up to 6 weeks to resolve. This delayed healing has been reported in a rabbit model where wounds created by PDT were slower to heal than those surgically produced (Meyer et al, 1991). Healing was delayed because of bone sequestrum in two dogs and bone necrosis in the other dog. Upon removal of the sequestrum and necrotic bone healing occurred. PDT has been touted as sparing normal tissue but the validity of the concept has been questioned (Grant et al, 1997). Applying PDT to the mandibles of normal rabbits produced no bone effects (Meyer et al, 1991). A possibility in our cases is that by destroying the tumour that was invading bone the blood supply was also destroyed. However, one of the dogs with a bone sequestrum did not have evidence of bone invasion. The peridontal ligament or its blood supply was another structure that was damaged by PDT. Normal teeth that were included in the treatment field became loose and were easily removed. Irradiation of normal teeth has been reported to have no effect (Meyer et al, 1991).

Two of the dogs that failed to have a sustained remission had large tumours. One of the dogs appeared to have remission with superficial healing occurring. The most likely explanation for failure in these cases is the failure to achieve light penetration to the complete tumour depth (Grant et al, 1997). The more aggressive nature of the larger tumours could also be responsible for failure of sustained remission because larger tumours also fail after surgical removal (Salisbury and Lantz, 1988). The third dog that failed treatment did so because of metastasis. Local control of the tumour appeared to be achieved. Metastasis in this case would likely have occurred with surgery or radiation therapy also.

PDT using HPPH as the photosensitizing agent was successful in the treatment of canine oral SCC that were smaller than 4 cm in diameter. The invasion of bone by the tumour did not affect the outcome. These results are similar to those obtained using Photofrin. Although the results may not be directly applicable to humans, previous work with PDT in dogs indicates that tumour response is similar to response in humans (Beck, 1992). Surgical debulking followed by PDT should be considered as a possible primary treatment in human oral SCC.

### REFERENCES

Beck ER (1992) Lasers in veterinary oncology. In: *Current Veterinary Therapy XI*, Bonagura JD (ed), pp. 414–418. WB Saunders: Philadelphia

Bellnier DA, Henderson BW, Pandey RK, Potter WR and Dougherty TJ (1993) Murine pharmacokinetics and antitumour efficacy of the photodynamic sensitizer 2-1 hexyloxyethyl-2-devinylpyropheophorbide-a. *J Photochem Photobiol B* **20**: 55–61

Boonkitticharoen V, Kulapaditharom B, Punnachaiya S and Kraiphibul P (1997) Differences in in vitro photodynamic sensitivity among head and neck cancers. *Lasers Med Sci* **12**: 274–279

Chang CJ, Lai YL and Wong CJ (1998) Photodynamic therapy for facial squamous cell carcinoma in cats using Photofrin. *Chang Keng I Hsueh* **21**: 13–19

Grant WE, Speight PM, Hopper C and Bown SG (1997) Photodynamic therapy: an effective, but non-selective treatment for superficial cancers of the oral cavity. *Int J Cancer* **71**: 937–942

Head KW (1990) Tumours of the alimentary tract. In: *Tumours in Domestic Animals*, Moulton JE (ed), pp. 347–428. University of California Press: Berkeley, CA

Magne ML, Rodriquez CO, Autry SA, Edwards BF, Theon AP and Madewell BR (1997) Photodynamic therapy of facial squamous cell carcinoma in cats using a new photosensitizer. *Lasers Surg Med* **20**: 202–209

Meyer M, Speight P and Bown SG (1991) A study of the effects of photodynamic therapy on the normal tissues of the rabbit jaw. *Br J Cancer* **64**: 1093–1097

Payne JT, McCaw DL, Casteel SW, Frazier D, Rogers K and Tompson RV (1996) Pharmacokinetics of pyropheophorbide-a-hexyl ether in the dog. *Lasers Surg Med* **18**: 406–409

Peaston AE, Leach MW and Higgins RJ (1993) Photodynamic therapy for nasal and aural squamous cell carconimas in cats. *J Am Vet Med Assoc* **202**: 1261–1265

Salisbury SK and Lantz GC (1988) Long-term results of partial mandibulectomy for treatment of oral tumours in 30 dogs. *J Am Anim Hosp Assoc* **24**: 285–294

Todoroff RJ and Brodey RS (1979) Oral and pharyngeal neoplasia in the dog: a retrospective survey of 361 cases. *J Am Vet Med Assoc* **175**: 567–571

Wenig BL, Kurtzman DM, Grossweiner LI, Mafee MF, Harris DM, Lobraico RV, Prycz RA and Appelbaum EL (1990) Photodynamic therapy in the treatment of squamous cell carcinoma of the head and neck. *Arch Otolaryngol Head Neck Surg* **116**: 1267–1270

© 2000 Cancer Research Campaign
